# Supplementary material for: Genomic Characteristics and E Protein Bioinformatics Analysis of JEV Isolates from South China from 2011 to 2018
Source: Vaccines (Basel). 2022 Aug 12;10(8):1303. doi: 10.3390/vaccines10081303 (PMC9412759; doi:10.3390/vaccines10081303)
Supplement: Supplementary file 1 [file vaccines-10-01303-s001.zip › vaccines-1819213-supplementary.pdf]

Supplement

Table S1. Referenced JEV strains used in this experiment

GenBank	strain	Genome length（bp）	Country	Year
MH165313.1	anheal	10965	China	2017
JF706270.1	BL06-50	10965	China	2006
JF706272.1	DL04-29	10977	China	2004
JF706273.1	Fj02-29	10977	China	2002
JN381867.1	Fj0276	10977	China	2002
JN381859.1	Fj0339	10977	China	2003
AF217620.1	FU	10964	Australia	1995
MF326270.1	GD	10977	China	2008
KF297915.1	GD-01	10976	China	2009
JN381843.1	GS07TS11	10965	China	2007
JF706274.1	GSBY0801	10965	China	2008
JN381857.1	GZ042	10977	China	2004
HM366552.1	GZ56	10965	China	2006
JF706276.1	HLJ02-134	10977	China	2002
JN381831.1	HN0411	10965	China	2004
JN381830.1	HN0621	10965	China	2006
AY184212.1	JKT6468	10978	China	1981
LC461957.1	MQ-amaguchi	10965	Japan	2016
HM596272.1	Muar	10988	Malaysia	1952
MH258849.1	SA14-14-2 MSV	10977	China	2018
JN381839.1	SC0412	10964	China	2004
JF706286.1	SD0810	10965	China	2008
JN381850.1	SH53	10965	China	2001
JN381856.1	SH0410	10977	China	2004
JN381847.1	SH03103	10965	China	2003
JF915894.1	XZ0934	10983	China	2009
HQ652538.1	XZ0938	10965	China	2009
JN381836.1	YN0623	10965	China	2006
JF706268.1	YN0967	10965	China	2009
JF706281.1	YN05124	10965	China	2005
KX779521.1	SC2016	10716	China	2016

LC623822.1	Mo-Kagawa	10915	Japan	2020
MT232844.1	Assam36	10965	India	2015
LC461960.1	Mindanao-K4	10976	China	2018
KR265316.1	Hubei	10976	China	2015
MH385014.1	YN	10976	China	2016
KP164498.2	IVRI395A	10976	India	2014
LC461961.1	Bali93	10970	Indonesia	2017
LC461958.1	THA185	10967	Thailand	2017
KU871354.1	BJ-1	10299	China	1995

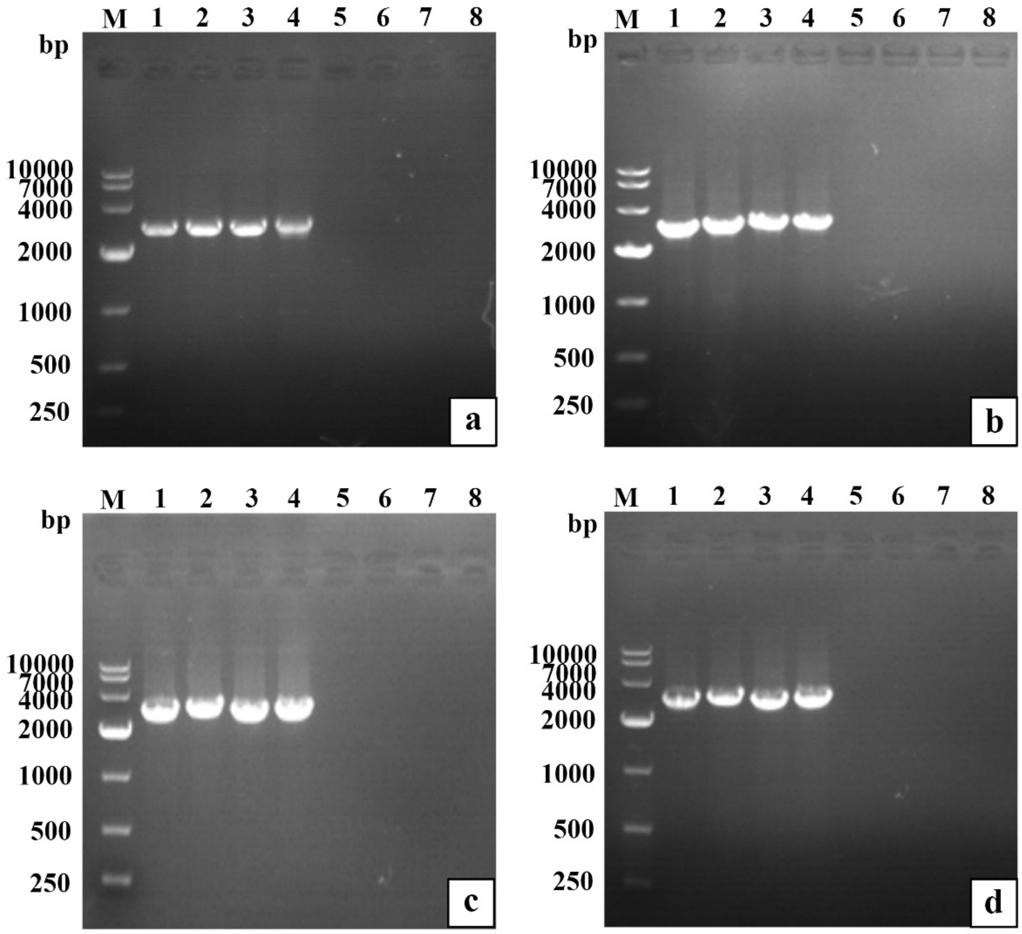

Figure S1: genomic amplification of four JEV isolates.a: CH/GD2011/2011 strains; b: CH/GD2014/2014 strains; c: CH/GD2015/2015 strains; d: CH/GD2018/2018 strains; M: DL2000 DNA marker; 1～4: Different JEV isolates were used as templates for amplification with JEV-P1-F/R Jev-P2-F /R Jev-P3-F /R and JEV-P4-F/R respectively; 5～8: ddH2O (negative control).

Table S2. Virus content assay of pigs JEV isolates

Strain	Virus titer （$TCID_{50}$/mL）								
	F1 ~ F2	F3	F4	F5	F6	F7	F8	F9	F10
CH/GD2015/2015	-	-	$10^{6.43}$	$10^{7.5}$	$10^{8.2}$	$10^{8.3}$	$10^{8.5}$	$10^{8.7}$	$10^{8.7}$
CH/GD2011/2011	-	-	$10^{6.17}$	$10^{7.3}$	$10^{8.0}$	$10^{8.17}$	$10^{8.0}$	$10^{8.17}$	$10^{8.17}$
CH/GD2014/2014	-	-	$10^{4.83}$	$10^{6.0}$	$10^{6.8}$	$10^{7.83}$	$10^{7.93}$	$10^{7.83}$	$10^{7.7}$
CH/GD2018/2018	-	-	$10^{4.9}$	$10^{5.9}$	$10^{6.7}$	$10^{7.3}$	$10^{7.5}$	$10^{7.7}$	$10^{7.5}$

Note: “-” means undetermined.

Table S3. Nucleotide sequence similarity analysis of the genes of JEV isolates

Gene	Nucleotide similarity （%）			
	CH/GD2011/2011	CH/GD2014/2014	CH/GD2015/2015	CH/GD2018/2018
Whole Gene	78.4 ~ 99.2	77.9 ~ 98.3	78.4 ~ 97.4	78.0 ~ 98.3
C	81.8 ~ 100.0	79.9 ~ 99.2	82.8 ~ 99.2	79.9 ~ 99.5
PrM	77.8 ~ 99.6	79.4 ~ 99.6	77.6 ~ 97.6	79.6 ~ 98.8
E	77.7 ~ 99.9	76.2 ~ 99.1	77.3 ~ 97.9	76.9 ~ 99.1
NS1	77.8 ~ 99.9	77.9 ~ 99.2	77.7 ~ 98.0	77.7 ~ 99.2
NS2a	76.9 ~ 100.0	76.9 ~ 99.3	77.8 ~ 98.5	76.4 ~ 98.7
NS2b	78.6 ~ 100.0	78.4 ~ 99.2	79.9 ~ 98.0	78.9 ~ 99.0
NS3	79.5 ~ 99.9	79.3 ~ 99.3	79.9 ~ 97.7	79.3 ~ 98.8
NS4a	77.8 ~ 100.0	77.2 ~ 99.2	77.0 ~ 98.1	77.0 ~ 99.2
NS4b	76.2 ~ 100.0	76.2 ~ 99.1	75.8 ~ 97.5	76.4 ~ 99.5
NS5	80.0 ~ 99.9	79.8 ~ 99.1	79.7 ~ 97.9	79.4 ~ 98.9

Table S4. Amino acid sequence similarity analysis of the gene of JEV isolates

Gene	Amino acid similarity （%）			
	CH/GD2011/2011	CH/GD2014/2014	CH/GD2015/2015	CH/GD2018/2018
C	81.3 ~ 100.0	79.7 ~ 99.2	82.1 ~ 98.4	78.9 ~ 99.2
PrM	88.6 ~ 99.4	89.8 ~ 100.0	88.0 ~ 98.2	89.8 ~ 100.0
E	89.6 ~ 99.8	91.2 ~ 100.0	91.2 ~ 99.2	91.2 ~ 100.0

NS1	90.6 ~ 100.0	91.2 ~ 100.0	91.5 ~ 99.7	91.2 ~ 100.0
NS2a	83.7 ~ 100.0	84.6 ~ 100.0	84.1 ~ 97.4	84.6 ~ 100.0
NS2b	92.4 ~ 100.0	90.8 ~ 99.2	93.9 ~ 100.0	91.6 ~ 100.0
NS3	94.0 ~ 99.8	94.5 ~ 100.0	94.7 ~ 99.8	94.5 ~ 100.0
NS4a	94.4 ~ 100.0	93.7 ~ 100.0	94.4 ~ 100.0	93.7 ~ 100.0
NS4b	91.4 ~ 100.0	91.0 ~ 100.0	91.8 ~ 100.0	90.6 ~ 100.0
NS5	91.8 ~ 99.9	92.0 ~ 99.8	92.0 ~ 99.8	92.2 ~ 100.0

Table S5 Common phosphorylation sites of E proteins of JEV isolates and their corresponding kinases

Site	Score	Kinase	Site	Score	Kinase	Site	Score	Kinase
T40	0.591	PKC	Y183	0.615	EGFR	S344	0.966	unsp
S51	0.564	DNAPK	Y183	0.599	unsp	T349	0.558	p38MAPK
T66	0.662	PKC	T187	0.738	unsp	T349	0.524	cdk5
T70	0.824	PKC	T187	0.552	CKII	T356	0.566	PKC
T76	0.844	unsp	S194	0.954	unsp	T363	0.747	PKC
T76	0.596	PKG	Y202	0.655	unsp	S364	0.571	unsp
S88	0.985	unsp	T205	0.853	PKC	S365	0.667	PKC
S88	0.56	PKA	S210	0.787	unsp	S368	0.785	unsp
S89	0.816	PKC	S227	0.871	unsp	Y382	0.973	unsp
Y90	0.95	unsp	S227	0.517	cdk5	Y382	0.553	INSR
Y90	0.506	EGFR	S230	0.586	PKC	S401	0.773	PKC
T97	0.702	PKC	S251	0.64	PKA	S401	0.565	cdc2
S112	0.586	PKA	S257	0.621	DNAPK	T402	0.786	PKC
T115	0.749	PKC	S257	0.532	unsp	S408	0.62	PKC
T122	0.902	PKC	Y301	0.518	SRC	S408	0.518	cdc2
Y137	0.503	unsp	T305	0.669	PKC	T409	0.537	cdc2
T147	0.665	unsp	S309	0.653	PKC	T410	0.854	unsp
T148	0.627	unsp	T317	0.918	unsp	T410	0.737	PKC
T148	0.548	PKC	S327	0.591	CKI	S429	0.618	CKI
S162	0.666	PKC	S329	0.996	unsp	T460	0.554	DNAPK
S162	0.604	DNAPK	T168	0.577	PKG	T460	0.55	cdc2
S162	0.539	ATM	S479	0.586	PKG	S479	0.784	PKA

Table S6 Different phosphorylation sites of E proteins of JEV isolates and their corresponding kinases

Strain	Site	Score	Kinase	Site	Score	Kinase	Site	Score	Kinase
CH/GD2015/2015	T232	0.823	unsp	S327	0.727	unsp	Y137	0.693	unsp
	T232	0.722	PKC	S329	0.528	cdc2	Y137	0.518	INSR
	S277	0.818	PKC	S88	0.985	unsp	S331	0.537	PKC
	S277	0.528	unsp	S88	0.56	PKA	S368	0.519	CKI
	T281	0.575	PKC	S89	0.816	PKC	T231	0.692	PKC
	S282	0.507	PKC	S129	0.57	PKA			
CH/GD2011/2011	S123	0.502	CKI	S329	0.528	cdc2	S331	0.537	PKC
	S257	0.521	ATM	S331	0.537	PKC	S368	0.519	CKI
	S327	0.727	unsp	S329	0.528	cdc2			
CH/GD2014/2014	S222	0.532	cdc2	T226	0.501	PKC	S277	0.528	unsp
	S222	0.531	DNAPK	S257	0.502	ATM	T281	0.575	PKC
	S222	0.518	PKA	S277	0.818	PKC	S282	0.507	PKC
	T226	0.504	GSK3						
CH/GD2018/2018	S222	0.532	cdc2	T226	0.501	PKC	S277	0.528	unsp
	S222	0.531	DNAPK	S257	0.502	ATM	T281	0.575	PKC
	S222	0.518	PKA	S277	0.818	PKC	S282	0.507	PKC
	T226	0.504	GSK3						

Table S7 B-cell antigenic epitopes of JEV isolate E protein

Strain	Number	B-cell epitope sequence	Starting point of amino acid position	Confidence value
CH/GD2011/2011 和 CH/GD2015/2015	1	GMSWITQGLMGALLLW	455	0.97
	2	YGEVTLDCEPRSGLNT	183	0.95
	3	DGPCKIPIVSVASLND	332	0.91
	4	VLEGDSCLTIMANDKP	24	0.91
	5	AALGDTAWDFGSIGGV	418	0.9
CH/GD2014/2014 和 CH/GD2018/2018	1	GMSWITQGLMGALLLW	455	0.97
	2	YGEVTLDCEPRSGLNT	183	0.95
	3	DGPCKIPIVSVASLND	332	0.91
	4	VLEGDSCLTIMANDKP	24	0.91
	5	AALGDTAWDFGSIGGV	418	0.9
	6	LSLPWTSPSSTAWRNR	221	0.9
